# Evaluation of Non-Uniform Image Quality Caused by Anode Heel Effect in Digital Radiography Using Mutual Information

**DOI:** 10.3390/e23050525

**Published:** 2021-04-25

**Authors:** Ming-Chung Chou

**Affiliations:** 1 Department of Medical Imaging and Radiological Sciences, Kaohsiung Medical University, Kaohsiung 80708, Taiwan; mcchou@kmu.edu.tw; 2 Center for Big Data Research, Kaohsiung Medical University, Kaohsiung 80708, Taiwan; 3 Department of Medical Research, Kaohsiung Medical University Hospital, Kaohsiung 80708, Taiwan

**Keywords:** circular-step wedge, contrast-detail, mutual information, visible ratio, anode heel effect

## Abstract

Anode heel effects are known to cause non-uniform image quality, but no method has been proposed to evaluate the non-uniform image quality caused by the heel effect. Therefore, the purpose of this study was to evaluate non-uniform image quality in digital radiographs using a novel circular step-wedge (CSW) phantom and normalized mutual information (nMI). All X-ray images were acquired from a digital radiography system equipped with a CsI flat panel detector. A new acrylic CSW phantom was imaged ten times at various kVp and mAs to evaluate overall and non-uniform image quality with nMI metrics. For comparisons, a conventional contrast-detail resolution phantom was imaged ten times at identical exposure parameters to evaluate overall image quality with visible ratio (VR) metrics, and the phantom was placed in different orientations to assess non-uniform image quality. In addition, heel effect correction (HEC) was executed to elucidate the impact of its effect on image quality. The results showed that both nMI and VR metrics significantly changed with kVp and mAs, and had a significant positive correlation. The positive correlation is suggestive that the nMI metrics have a similar performance to the VR metrics in assessing the overall image quality of digital radiographs. The nMI metrics significantly changed with orientations and also significantly increased after HEC in the anode direction. However, the VR metrics did not change significantly with orientations or with HEC. The results indicate that the nMI metrics were more sensitive than the VR metrics with regards to non-uniform image quality caused by the anode heel effect. In conclusion, the proposed nMI metrics with a CSW phantom outperformed the conventional VR metrics in detecting non-uniform image quality caused by the heel effect, and thus are suitable for quantitatively evaluating non-uniform image quality in digital radiographs with and without HEC.

## 1. Introduction

Image quality is an essential requirement in digital X-ray imaging and is closely associated with the accuracy of disease diagnosis. The fundamental metrics of static image quality are contrast, spatial resolution, and noise, which can be evaluated through the measurements of modulation transfer function (MTF), point-spread function, and noise power spectrum (NPS) [1,2,3]. Although these metrics can be measured from an X-ray imaging system, the individual metrics cannot correctly reflect the overall image quality. Detective quantum efficiency (DQE), which is a function of MTF, NPS, and system gain, is the most commonly used metric to quantify the overall performance of X-ray imaging systems [4,5,6]; however, DQE cannot reflect entire imaging chains, such as image processing and correction [7]. In contrast, a more practical approach to quantifying overall image quality of a radiograph is to use contrast-detail phantoms [8,9,10,11,12]. Previously, an emerging metric, termed as mutual information (MI), was shown to successfully quantify the overall image quality of a digital radiograph with the use of a linear step-wedge phantom [13,14]. Although these metrics were shown to be capable of quantifying overall image quality, none are suitable for evaluating the non-uniform image quality of an image caused by the anode heel effect.

In radiography, the “heel effect” causes less X-ray fluence and higher mean radiation energy in the anode direction due to the absorption of low-energy photons by the anode heel [15]. The non-uniform distribution of X-ray fluence may result in non-uniform image quality, especially in the anode-cathode direction. However, there were limited previous works quantifying the influence of anode heel effect on image quality in digital radiographs [16]. Previous studies demonstrated that the heel effect significantly impacted the signal-to-noise ratio (SNR) using an anthropomorphic phantom, but the image quality was not significantly different between pelvic radiographs with the head towards the anode and cathode directions [17,18]. Moreover, some previous studies performed post-processing heel effect correction (HEC) to minimize the inhomogeneous intensity in radiographs [19,20,21]. However, no suitable method has been presented that can objectively quantify the non-uniform image quality in radiographs. Moreover, no methods can elucidate how much the image quality can be improved in the radiographs with HEC. Therefore, the purposes of this study were three-fold: (1) to design a circular step-wedge (CSW) phantom for evaluating overall and non-uniform image quality, (2) to compare other image quality metrics measured from a contrast-detail phantom, and (3) to understand how much HEC can improve the image quality.

## 2. Materials and Methods

### 2.1. Circular Step-Wedge Phantom

In information theory, MI is a measure of mutual dependence between two random variables, and is calculated from their individual entropy and joint entropy, defined as

**Figure 1:**
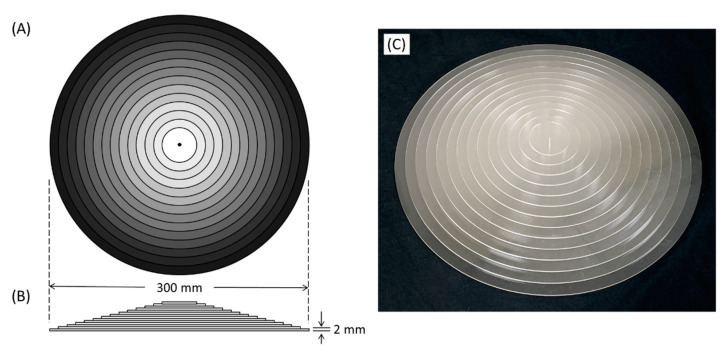
The top view (**A**), lateral view (**B**), and actual image (**C**) of the CSW phantom consisting of 14 steps with 2 mm incremental thickness (from 2 to 28 mm), and 20 mm incremental diameter (from 40 to 300 mm).

MI = H(X) + H(Y) − H(X, Y),

where H(X) and H(Y) are individual entropy of random variables (X and Y), and H(X, Y) is their joint entropy [22]. As MI reflects the amount of information of one random variable that is observed from the other random variable, it is possible to utilize the MI metrics to reflect the image quality using a linear step-wedge phantom [13,14]. However, the original design can only measure MI in one direction parallel to the long axis of the phantom, so it is unable to evaluate non-uniform image quality in radiographs caused by anode heel effect. Therefore, the present study designed a CSW phantom with acrylic material to estimate the MI metrics in different directions from a single image. The phantom was fabricated using 14 pieces of circular acrylic board with radii from 4 cm to 30 cm, which were precisely (±0.1 mm) laser cut from a 2 mm thick acrylic plastic sheet. After a 1 mm hole (diameter) was drilled in the center, 14 circular acrylic boards were piled up sequentially from large to small and were aligned and glued together at the center. The CSW phantom consisted of 14 steps with thickness from 2 mm to 28 mm and with radii from 4 cm to 30 cm, as shown in Figure 1.

### 2.2. Contrast-Detail Resolution Phantom

**Figure 2:**
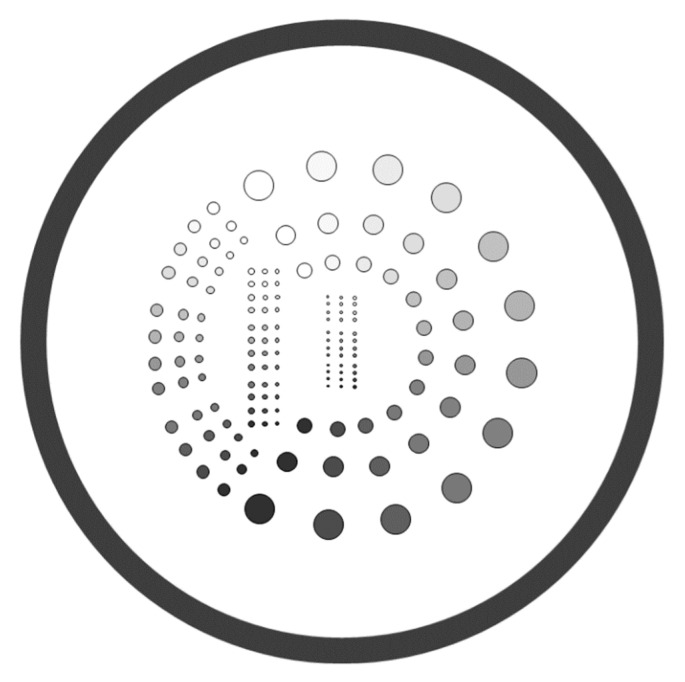
The arrangement of 144 disc details within the TO16 CDR phantom. In the phantom, 72 larger disc details are arranged circularly in the outer region, and 72 smaller ones are arranged linearly in the central region.

A commercial contrast-detail resolution (CDR) phantom was also used to evaluate the overall image quality of radiographic images. The phantom consists of 144 circular details with 12 sizes × 12 contrasts (TO16, Leeds Test Objects LTD, North Yorkshire, UK; https://www.leedstestobjects.com (accessed on 30 March 2021)) [9]. Of the 144 details, 72 larger details were arranged circularly in the outer region, and the remaining 72 smaller details were arranged linearly in the central region, as shown in Figure 2.

### 2.3. Image Data Acquisition

Image quality was evaluated using both CSW and CDR phantoms in a digital radiographic system (Toshiba/DRX-3724HD) that was equipped with a CsI flat panel detector (a-Si, TFT, CXD-70C wireless). The X-ray images were acquired from the two phantoms with matrix size = 2800 × 3408, pixel size = 0.13 × 0.13 mm^2^, dynamic range = 4096, and source-to-detector distance = 100 cm. For statistical analysis, image acquisition was repeated ten times at 40, 45, 50, 55, and 60 kV (5 mAs), and at 5, 10, 20, 25, and 40 mAs (40 kVp), respectively. A posterior-anterior right-hand radiograph was acquired with 52 kVp and 10 mAs to show the impact of anode heel effect on image quality. The human study was approved by the local institutional review board (KMUHIRB-E(I)-20200274).

### 2.4. Mutual Information with a CSW Phantom

**Figure 3:**
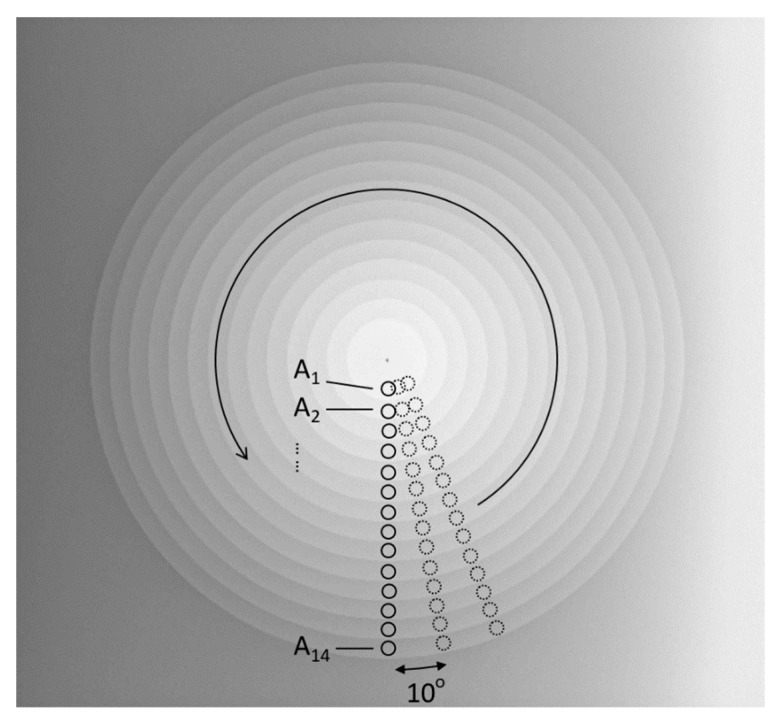
The estimation of the nMI metrics in 36 orientations separated by 10 degrees. 14 equal-sized circular ROIs (A1 to A14) are placed respectively on the step centers to calculate the nMI metrics. Afterwards, the 14 ROIs are rotated counterclockwise by multiples of 10 degrees to estimate the corresponding nMI metrics in other orientations.

This study estimated MI from an X-ray image of the CSW phantom using a home-made script on a MATLAB software. First, the center of the CSW phantom in the image was detected by the center of gravity. Second, 14 circular regions-of-interest (ROIs), each containing 1941 pixels, were automatically placed on the center of 14 steps, respectively, in one direction, as shown in Figure 3. Subsequently, the 14 ROIs were rotated counterclockwise around the center every 10 degrees, from which 36 MI metrics were calculated. For each direction, the MI metrics were calculated according to the method reported by previous studies [13,14]. However, since a larger number of steps of the phantom would give rise to larger MI values (bits), the present study calculated a normalized MI (nMI) [23,24], defined as MI/log2(N) × 100 %. N is the number of steps in the CSW phantom. The resultant nMI ranges from 0 to 100%, and a larger nMI value indicates better image quality.

### 2.5. Visible Ratio with a CDR Phantom

This study measured visible ratio (VR) metrics using a TO16 CDR phantom with a commercial AutoPIA tool (Leeds Test Objects LTD, North Yorkshire, UK). The phantom was rotated counterclockwise every 30 degrees from 0 to 180 degrees to understand whether the CDR phantom can adequately reflect the anode heel effect on image quality. For each orientation, ten repeated X-ray images of CDR phantoms were acquired for comparisons and were analyzed automatically to detect all possible details. In this step, the software calculated the contrast-to-noise ratio (CNR) for each of 144 details, defined as |(target signal − background signal)|/(background noise), and then those details with CNR higher than a predefined threshold were considered as visible details [9]. Finally, the VR metrics, defined as (number of successfully detected details)/(total number of details) × 100 %, were calculated to give a value between 0 to 100%. Similarly, a larger VR metrics indicates better image quality and higher performance in detecting details.

### 2.6. Heel Effect Correction

**Figure 4:**
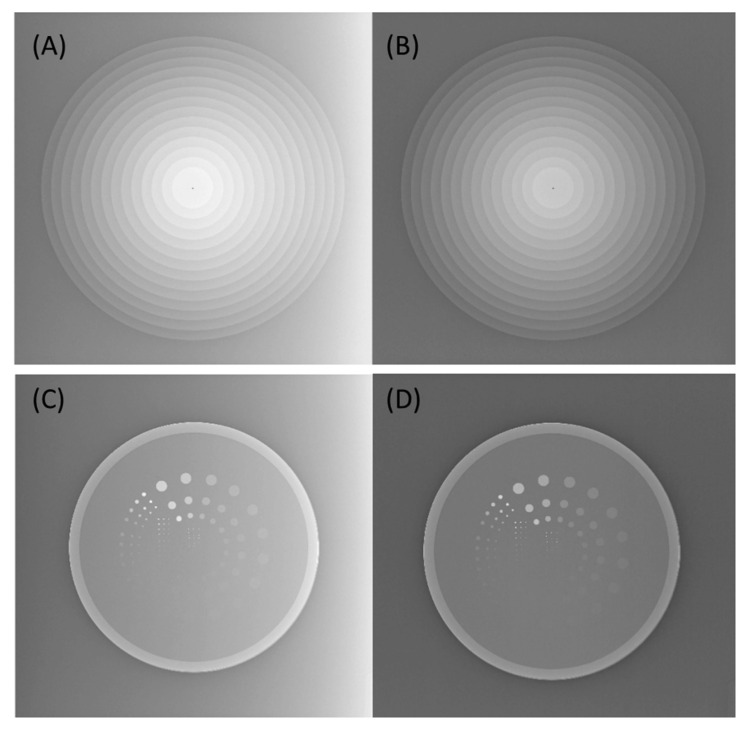
The CSW (**A**) and CDR (**C**) images acquired with 40 kVp and 5 mAs exhibited inhomogeneous signal intensity in the anode-cathode (horizontal) direction due to the heel effect. The inhomogeneity was successfully removed in the corrected CSW (**B**) and CDR (**D**) images after HEC.

This study performed a retrospective correction method that minimizes the intensity inhomogeneity in the X-ray images by fitting the background signals to a 2nd order polynomial function in the anode-cathode direction to understand how the HEC impacts the image quality. Subsequently, the phantom image was subtracted by the fitted curve and added by a minimum value of the curve to keep similar image brightness, as shown in Figure 4. Finally, nMI and VR metrics were estimated from the phantom images with and without HEC.

### 2.7. Statistical Analysis

A one-way analysis of variance (ANOVA) was performed to understand whether the image quality metrics significantly changed with kVp, mAs, and orientations before and after HEC, respectively. A post-hoc Mann–Whitney U test was used to compare the differences between two exposure parameters and between two orientations. The Wilcoxon signed rank test was conducted to show the difference in nMI and VR metrics before and after HEC [25]. Moreover, Pearson’s correlation analysis was carried out to reveal the relationship between the two metrics before and after HEC, respectively [26]. Statistical significance (P) was deemed if P < 0.05.

## 3. Results

**Figure 5:**
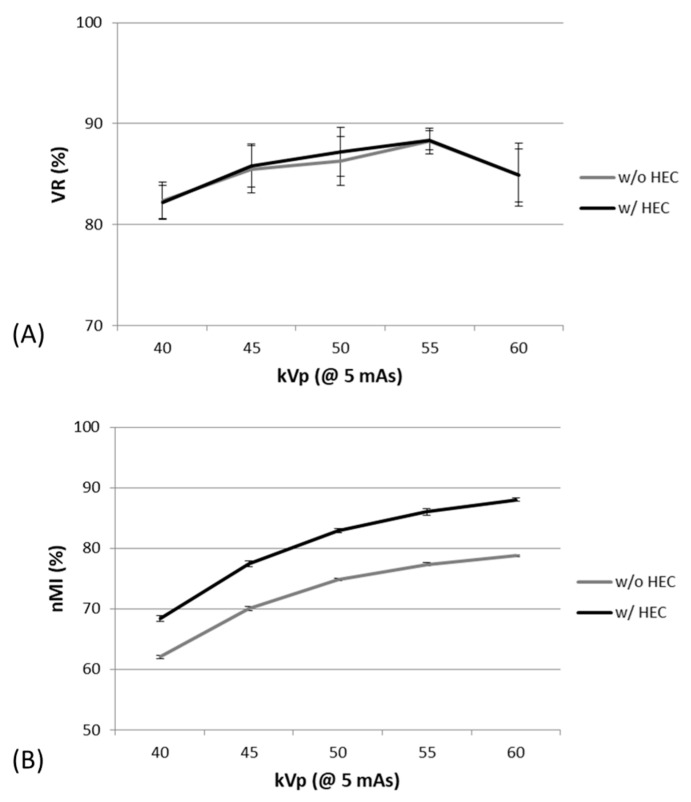
The VR (**A**) and nMI (**B**) metrics changed significantly with kVp, at 5 mAs, before and after HEC.

By varying kVp, one-way ANOVA analysis showed that both nMI and VR metrics significantly changed with kVp between 40 and 60 kVp at a constant 5 mAs. It was also found that nMI changed more prominently than VR in X-ray images with and without HEC, as shown in Figure 5. The Mann–Whitney U test highlighted a significant difference in nMI metrics between any two kVps; however, the VR metrics were not significantly different between 45 to 50 kVp, 45 to 60 kVp, or 50 to 60 kVp in images with and without HEC. Moreover, the nMI metrics were significantly increased after HEC; however, no significant change was noted in the VR metrics at different kVps after HEC.

**Figure 6:**
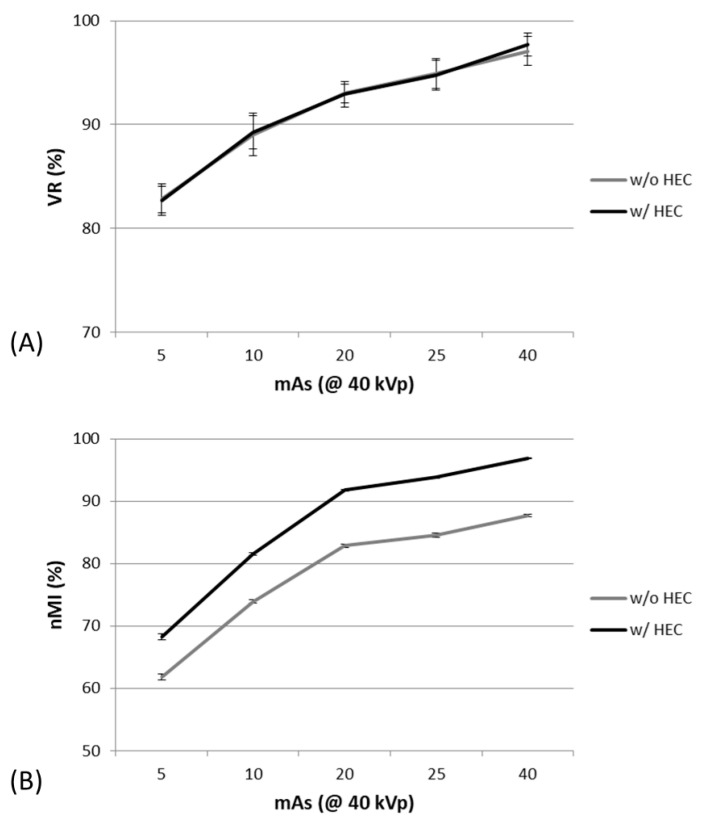
The VR (**A**) and nMI (**B**) metrics changed significantly with mAs, at 40 kVp, before and after HEC.

By varying mAs, one-way ANOVA analysis showed that both nMI and VR metrics also significantly changed with mAs between 5 and 40 mAs at a constant 40 kVp, as shown in Figure 6. 

**Figure 7:**
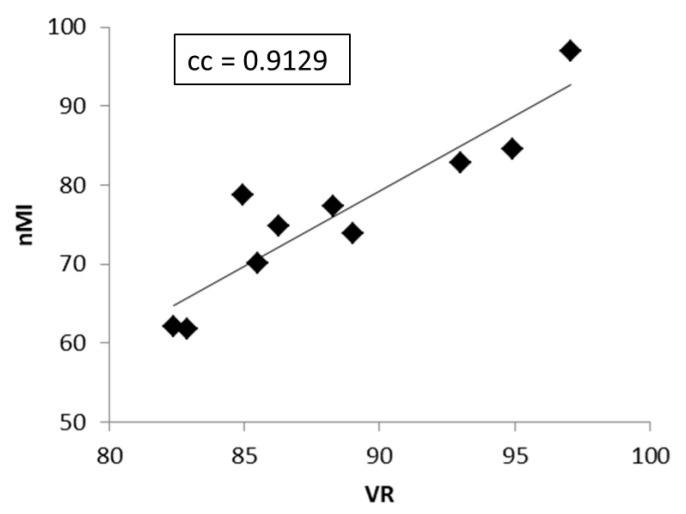
The significant correlation (cc = 0.9129, P < 0.05) between the VR and nMI metrics measured from all exposure parameters in images without HEC.

The post-hoc Mann–Whitney U test showed that both nMI and VR were significantly different between any two mAs in the images with and without HEC. The nMI metrics were significantly increased after HEC; however, no significant change was noted in the VR metrics at any of the mAs after HEC. Moreover, the averaged nMI and VR metrics significantly correlated in the images without HEC, as shown in Figure 7.

**Figure 8:**
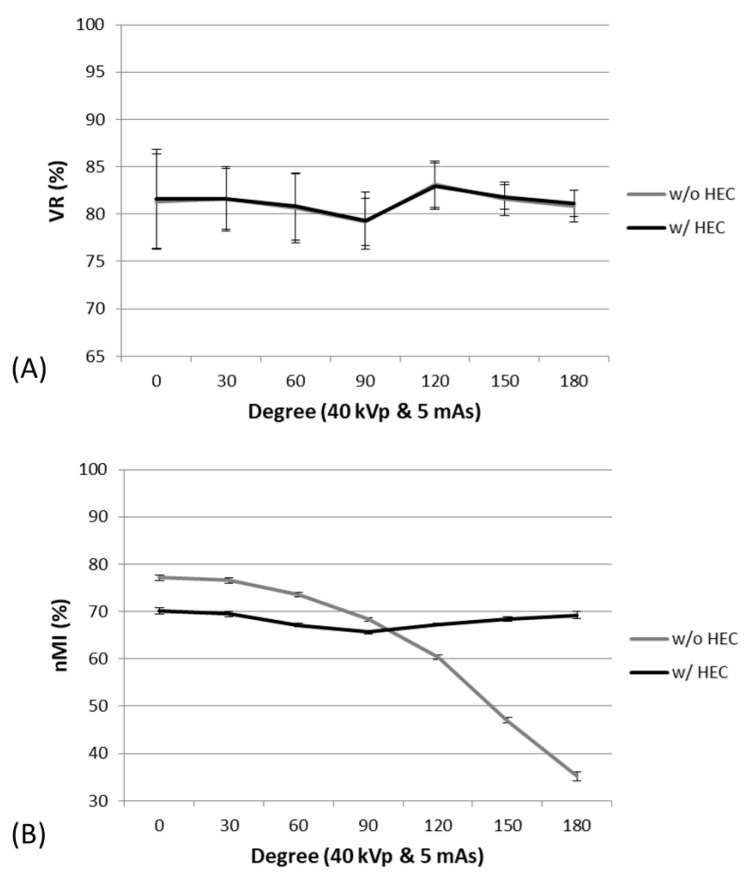
The VR (**A**) and nMI (**B**) metrics measured as a function of orientation at 40 kVp and 5 mAs. The VR metrics did not change significantly with orientations, whereas the nMI metrics changed significantly with orientations in the images without HEC.

By varying orientation in the measurement, without HEC, there were significant changes in nMI with orientations between 0 and 180 degrees (the results were symmetric around 180 degrees). However, without HEC, there were no significant changes in VR with orientations between 0 and 180 degrees, as shown in Figure 8. The post-hoc Mann–Whitney U test showed that the nMI metrics were significantly different between two orientations in images with and without HEC. Although the nMI metrics came to be more uniform across different orientations, there remains slight difference in nMI metrics between 30 and 150 degrees.

**Figure 9:**
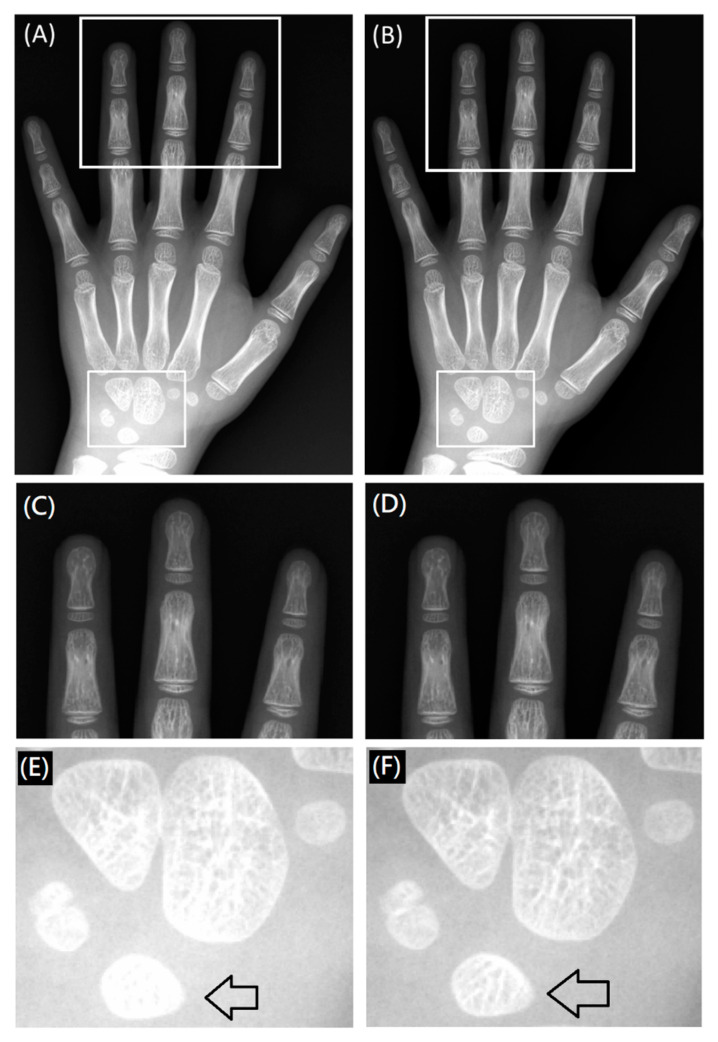
A posterior-anterior right-hand image acquired with 52 kVp and 10 mAs before (**A**,**C**,**E**) and after (**B**,**D**,**F**) HEC. The arrows indicate the bony structures of the lunate that were more conspicuous in the image with (**F**) than without (**E**) HEC.

A posterior-anterior right-hand X-ray image (Figure 9) demonstrated inhomogeneous signal intensity in the anode-cathode direction due to the heel effect, where lower signal intensity (higher X-ray exposure) was noted in the finger than the wrist direction (Figure 9A,C,E). By applying the HEC, the inhomogeneity issue was minimized across the entire image, and small bony structures were more conspicuous in the corrected image than the raw image displayed with an identical window level and width (Figure 9B,D,F). Although the bony structures of the wrist in the raw image can be visualized by adjusting the window level and width, the bony structures of the fingers will be too dark to be visualized. This inhomogeneous issue can be reflected by the inconsistent nMI metrics in radial direction, as shown in Figure 8.

## 4. Discussion

In radiography, the heel effect causes less X-ray fluence and higher mean radiation energy in the anode direction, and results in non-uniform image quality. Although there have been some methods proposed to reduce the heel effect [19,20,21], no suitable method has been presented that can objectively quantify the overall and non-uniform image quality caused by the heel effect. This study designed a CSW phantom for quantification of overall and non-uniform image quality in X-ray radiographs using nMI metrics based on information theory. The nMI metrics were demonstrated to be associated with imaging SNR, contrast, and resolution [13,14]. In the present study, the evaluated image quality was compared between the nMI (CSW phantom) and conventional VR (CDR phantom) metrics in digital X-ray images acquired at various exposure parameters and orientations, and with and without HEC. The results highlight that both metrics significantly changed with kVp (from 40 to 60 kVp at 5 mAs) and mAs (from 5 to 40 mAs at 40 kVp). The overall image quality assessed by nMI and VR metrics exhibited a similar trend with high correlation, suggesting that both metrics are capable of reflecting image quality in digital X-ray images. In addition, the nMI metrics were found to be more sensitive to changes in exposure parameters (kVp and mAs) than the VR metrics. It is postulated that the increased sensitivity is due to the fact the CSW phantom was made of acrylic material and had a small difference in thickness. 

It is known that the anode heel effect may lead to heterogeneous X-ray exposure that can deteriorate overall image quality. The results of the present study demonstrated that the heel effect significantly deteriorated the overall image quality. Furthermore, the image quality reflected by the nMI metrics can be significantly improved with HEC in the anode direction; this correction resulted in improved homogeneity of image quality and higher conspicuity of bony structures in the hand X-ray images. However, the conventional VR metrics were not significantly changed with orientations before and after HEC, suggesting that the nMI metrics were more sensitive than the VR metrics to non-uniform image quality.

The insensitivity of VR metrics to detect non-uniformity of image quality was likely attributable to the fact that the disk details were embedded in the central area of the CDR phantom, as shown in Figure 2. Although the centralized disk details in the CDR phantom were suitable for measuring the image quality in the central field of view, the design itself rendered it less sensitive to inhomogeneous image quality that occurred in the outer region. On the contrary, the nMI metrics were calculated from the image of CSW phantom made of acrylic material and with a suitable size that fits the flat panel detector. A previous study showed that the image quality reflected by the correctly identified holes (%) of the CDRAD phantom was more sensitive to changes in exposure parameters than the number of detected details in a CDR phantom [12], suggesting that the acrylic material of the CDRAD phantom was sensitive to changes in signal intensity. Similarly, our results demonstrated that the nMI metrics (CSW phantom) were more sensitive to changes in exposure parameters and orientations than the VR metrics (CDR phantom). The results indicated that the nMI with the CSW phantom could potentially be a quantifiable metric for non-uniform image quality in digital X-ray images.

Some limitations, however, warrant discussion. First, a small range of exposure parameters was utilized in this study. A study using a broader range of exposure parameters may provide more comprehensive comparisons between the two metrics. Second, the nMI metrics with the CSW phantom have an intrinsic disadvantage of less sensitivity to changes in spatial resolution [13]. However, the circular nature of CSW phantom can be used to estimate radial MTF, as proposed by a previous study [27], so in addition to nMI, the CSW phantom can be utilized to evaluate the radial MTF in X-ray images. Third, the CSW phantom was designed with acrylic material, so it may not be suitable to measure the image quality at high kVp and high mAs. A CSW phantom with a combination of aluminum and acrylic materials may be helpful to reflect image quality of X-ray images acquired using clinical parameter settings. Further investigations will be needed to compare the results between phantoms made of different materials.

## 5. Conclusions

In conclusion, the nMI with the CSW phantom performs as well as VR does with the CDR phantom in evaluating overall image quality in digital X-ray images. Moreover, both metrics had a significantly high correlation at various exposure parameters. The nMI metrics further outperformed the VR metrics in detecting heel effects associated with non-uniform image quality. The nMI metrics also had higher sensitivity to changes in image quality after HEC. Therefore, we concluded that the proposed nMI metrics with the CSW phantom are suitable for evaluating overall and non-uniform image quality in digital X-ray images.

## Data Availability

The data presented in this study are available on request from the corresponding author.

## Abbreviations

CSW	Circular Step-Wedge
CDR	Contrast-Detail Resolution
HEC	Heel Effect Correction
MTF	Modulation Transfer Function
nMI	normalized Mutual Information
NPS	Noise Power Spectrum
ROI	Region of Interest
VR	Visible Ratio
